# Association between small dense low-density lipoprotein cholesterol and neuroimaging markers of cerebral small vessel disease in middle-aged and elderly Chinese populations

**DOI:** 10.1186/s12883-021-02472-6

**Published:** 2021-11-09

**Authors:** Xiaorong Yu, Yanhua Yu, Cunsheng Wei, Lin Wang, Junying Jiang, Rui Zhang, Qi Dai, Yue Kang, Xuemei Chen

**Affiliations:** Department of Neurology, The Affiliated Jiangning Hospital with Nanjing Medical University, Nanjing, 211100 Jiangsu China

**Keywords:** LDL subclasses, Small and dense LDL, Cerebral small vessel disease, Burden, MRI markers

## Abstract

**Background:**

Cerebral small vascular disease (CSVD) is one of the leading causes of death in the aged population and is closely related to abnormalities in low-density lipoprotein cholesterol (LDL-C). Our study aims to clarify the relationship between small and dense low-density lipoprotein cholesterol (sdLDL-C) (a subcomponent of LDL-C) and neuroimaging markers of CSVD.

**Methods:**

In total, 1211 Chinese adults aged ≥45 years with cranial magnetic resonance imaging (MRI) were recruited in this retrospective study from January 2018 to May 2021. Serum lipids and other baseline characteristics were investigated in relation to the occurrence of CSVD. A logistic regression model was performed to analyze the relationships between LDL subtypes and CSVD risk, and the Pearson correlation coefficient was used to analyze the correlation between clinical characteristics and CSVD risk. ROC curves and AUCs were created and depicted to predict the best cutoff value of LDL-C subtypes for CSVD risk. Based on these data, we performed comprehensive analyses to investigate the risk factors for CSVD.

**Results:**

Ultimately, 623 eligible patients were included in the present study. Of the 623 eligible patients, 487 were included in the CSVD group, and 136 were included in the group without CSVD (control group). We adjusted for confounders in the multivariate logistic regression model, and LDL-C3 was still higher in the CSVD patients than in the group of those without CSVD (OR (95% CI), 1.22(1.08–1.38), *P* < 0.05). Pearson correlation showed that there was a positive correlation between the levels of LDL-C3, LDL-C4, LDL-C5, glucose, age, hypertension, previous ischemic stroke and CSVD risk (*r >* 0.15, *P <* 0.01). Moreover, the best cutoff value of LDL-C3 to predict CSVD was 9.5 mg/dL with 68.4% sensitivity and 72.8% specificity, and the best cutoff value of LDL-C4 to predict CSVD was 5.5 mg/dL with 50.5% sensitivity and 90.4% specificity.

**Conclusion:**

The results indicate that LDL-C3 is an independent risk factor for CSVD. A new prediction model based on LDL-C3 and LDL-C4 can help clinicians identify high-risk CSVD, even in people with normal LDL-C levels. The levels of sdLDL-C should be considered in the assessment and management of CSVD.

**Supplementary Information:**

The online version contains supplementary material available at 10.1186/s12883-021-02472-6.

## Background

Cerebral small vascular disease (CSVD) is an imaging, pathological and clinical syndrome caused by intracranial small vessel disease [1, 2], and it shows a high prevalence in the aged population [2–4]. CSVD is one of the leading causes of stroke outcome [5–7], cognitive impairment [8, 9], and several neurological symptoms [10, 11]. Cranial magnetic resonance imaging (MRI) markers of CSVD include white matter hyperintensity (WMH), lacunes, lobar and deep cerebral microbleeds (CMBs), and enlarged perivascular spaces (PVSs) [1]. These MRI markers might reflect the vulnerability of individual brains to pathologic insults. Therefore, it is necessary to screen the risk factors associated with MRI markers of CSVD.

Atherosclerosis is well known to contribute to the occurrence and development of ischemic stroke and CSVD [12–14], which is closely associated with abnormalities in serum lipids and lipoproteins, including elevated triglyceride levels, reduced high-density lipoprotein (HDL) cholesterol concentrations and increased low-density lipoprotein cholesterol (LDL-C) concentrations [15]. Specifically, serum LDL-C elevation is a common manifestation of dyslipidemia and leads to atherosclerosis [15, 16]. It is also an important risk factor for CSVD. Small and dense low-density lipoprotein cholesterol (sdLDL-C) is a subcomponent of LDL-C with small particles and high density and includes LDL-C3 to LDL-C7 [16]. It is easily oxidized, cleared slowly and enters the arterial wall to cause atherosclerosis, which is harmful [17].

Previous studies have shown that sdLDL-C can be used as an independent marker of coronary heart disease [18, 19]. The risk assessment level of established risk factors is even better than that of traditional risk factor markers. In the CNS, sdLDL-C was associated with an increased risk of acute ischemic stroke (AIS), especially noncardioembolic stroke [19]. In addition, sdLDL-C was related to AIS severity and prognosis, suggesting the importance of sdLDL-C control in patients with neurological diseases [20]. However, little is known about the relationship between sdLDL-C and CSVD to further explore whether sdLDL-C plays a key role in the early stage of atherosclerosis in CSVD. If sdLDL-C is involved, the question arises as to whether it is a better indicator to predict the risk of CSVD in the general population.

Therefore, we aimed to undertake the present study to clarify the association between sdLDL-C and neuroimaging markers of CSVD.

## Methods

### Study population

**Fig. 1:**
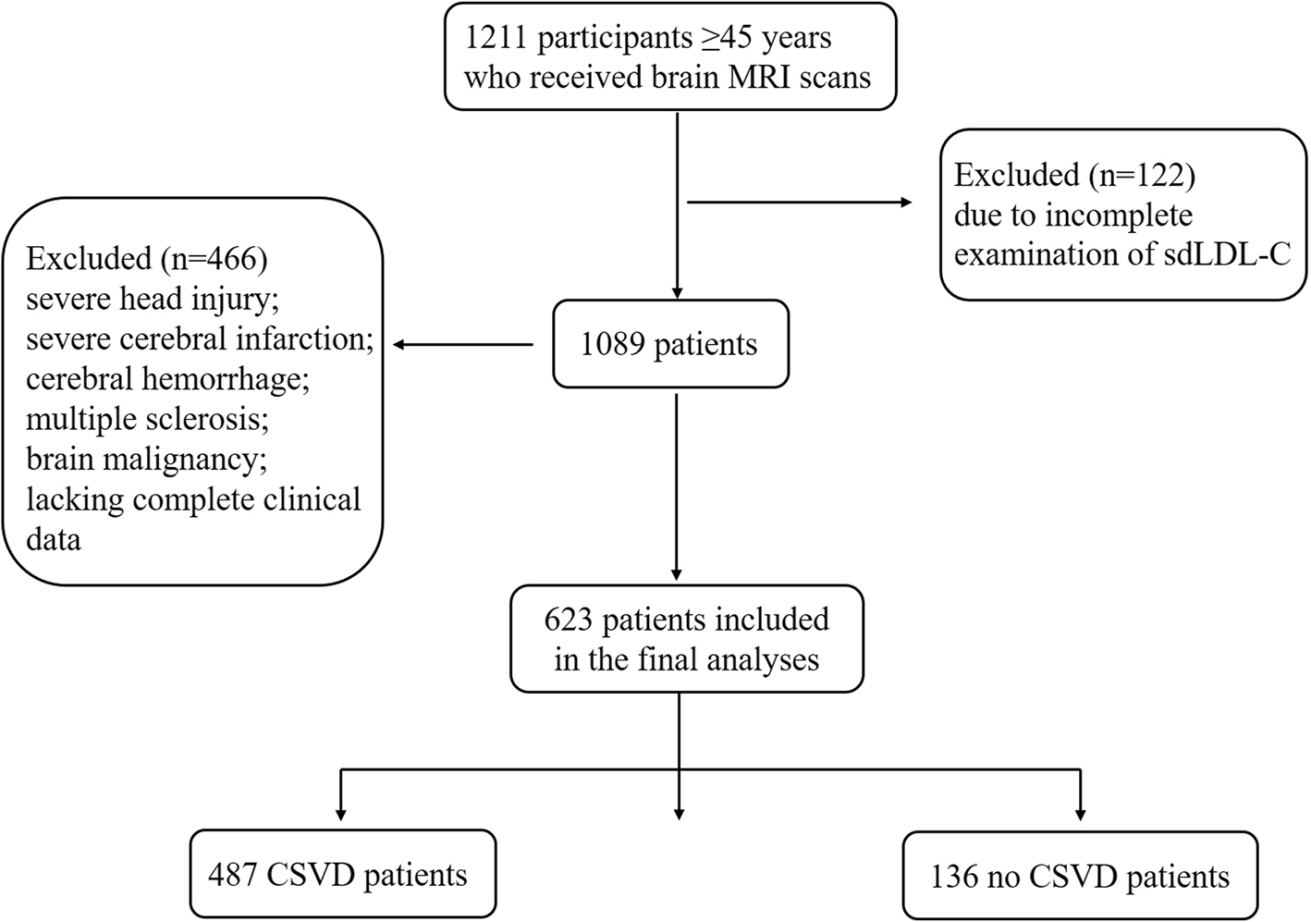
Diagrammatic sketch of the screening process

The study group consisted of 1211 hospitalized patients due to dizziness or headaches from the Department of Neurology, the Affiliated Jiangning Hospital of Nanjing Medical University, between January 2018 and May 2021. The inclusion criteria were as follows: Aged ≥45 years and cerebral MRI. The exclusion criteria were as follows: Participants with indications that may affect the evaluation of CSVD and those with severe head injury, defined as a head Abbreviated Injury Score (AIS) > 4 [21]; severe cerebral infarction was defined as the NIH Stroke Scale (NIHSS) ≥ 14 [22]; severe cerebral hemorrhage, defined as baseline intraparenchymal hemorrhage volume ≥ 30 ml or intraventricular hemorrhage [23]; or presence of multiple sclerosis, lacking complete clinical data, or brain malignancy. The study flowchart is shown in Fig. 1. Each subject signed the informed consent. The study protocols were approved by the Ethics Committee of Affiliated Jiangning Hospital of Nanjing Medical University.

### MRI acquisition

MRI scanning was performed on a 3.0 T MRI scanner (Ingenia, Philips Medical Systems, the Netherlands) with an 8-channel receiver array headcoil. High-resolution T1-weighted axial images covering the whole brain were obtained by a 3D magnetization prepared rapid gradient-echo sequence: TR = 8.1 ms; FA = 90°; TE = 3.7 ms; FOV =240 × 240 mm; acquisition matrix = 240 × 222; gap = 0 mm, thickness = 1.0 mm; number of slices = 170. T2-weighted images were acquired using the following parameters: TR = 4000 ms; TE =107 ms; FA = 90°; FOV =230 × 230 mm acquisition matrix = 384 × 384; thickness = 1.5 mm; gap = 0 mm, number of slices = 18. DWI-weighted images were obtained with the following: TR = 2503 ms; TE =98 ms; FA =90°; FOV =230 × 230 mm acquisition matrix =152 × 122; thickness = 1.5 mm; gap = 0 mm, number of slices = 18. Susceptibility weighted imaging (SWI)-weighted images were acquired using the following parameters: TR = 16 ms; TE =23 ms; FA =10°; FOV =220 × 180 mm; acquisition matrix = 220 × 180; thickness = − 0.6 mm; gap = 0 mm, number of slices = 200. Additionally, T2 fluid-attenuated inversion recovery (FLAIR) axial images were obtained with the following parameters: TR = 10,000 ms; TE = 120 ms; FA =110°; FOV =220× 220 mm; acquisition matrix = 336 × 189; thickness = 1.5 mm; gap = 0 mm, number of slices =18 [24].

### Fazekas score and CSVD burden score

**Fig. 2:**
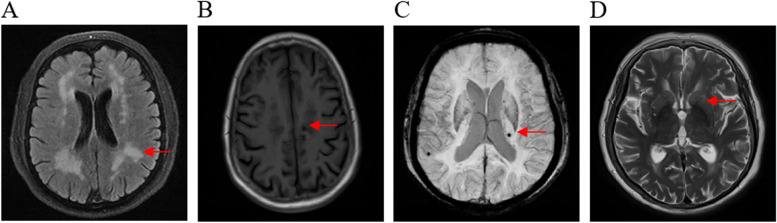
MRI manifestations of cerebral small vessel disease

The results are reported in accordance with STRIVE [25]. The Fazekas score was used to score WMH [26]. We calculated the total CSVD score [27–29] using an ordinal scale (0 to 4) and counting the presence of each of the four MRI markers for CSVD (Fig. 2). Briefly, one point was awarded for each of the following items: Moderate to extensive (10–25 or > 25) PVS in the basal ganglia (1 point if present); ≥1 lacune (1 point if present); periventricular WMH Fazekas score 3 or if deep WMH Fazekas score 2 or 3 (1 point if present); and ≥ 1 deep CMB (1 point if present) [30]. The patients were divided into two groups according to their total CSVD score, that is, with CSVD (1–4 points) or without CSVD (0 points).

### Data collection

The risk factors for cerebrovascular diseases were collected from each subject, including clinical and demographic information. The information consisted of age, sex, body mass index (BMI), medical history, and past or present cigarette or alcohol use. In addition, laboratory tests were carried out to determine fasting blood glucose (FBG), glycosylated hemoglobin (HbA1c), D-dimer levels, pulse pressure difference, total cholesterol (TC), triglyceride (TG), low-density lipoprotein (LDL), lipoprotein a [Lp(a)], lipoprotein-associated phospholipase A2 (Lp-PLA2), high-sensitivity C-reactive protein (H-CRP), uric acid (UA), and creatinine (Cr). All the samples were collected from the venous blood of the subjects in a 12-h fasted state.

### Low-density lipoprotein analysis

Blood samples were also used for LDL subfraction analysis. LDL subgroups were classified and measured with the Lipoprint System (Quantimetrix Corporation, Redondo Beach, CA, USA) according to the manufacturer’s instructions as previously described [31, 32]. This system separates LDL subfractions by a polyacrylamide gel electrophoresis technique, and gels were scanned to determine the relative area of each lipoprotein subfraction after electrophoresis. Based on net surface charge and size, various stained LDL subfractions are classified according to their mobilities in the gel. By this analysis, LDL was divided into 7 subfractions (LDL-1 to LDL-7) [33]. LDL-1 and LDL-2 are defined as larger LDLs, and LDL3–7 are defined as sdLDL-C according to their size and density.

### Statistical analysis

SPSS software (Version 22.0) was applied to analyze the data. Categorical variables are shown as numbers combined with percentages (%). Continuous variables are shown as the mean ± standard deviation (SD). The correlation of clinical variables with the risk of CSVD was analyzed by Pearson correlation analysis. For the univariate analysis, differences in clinical data were analyzed by Student’s *t*-test and chi-square tests. To adjust for traditional risk factors, multivariable logistic regression analysis was performed by binary logistic regression analysis, which allowed adjustment for confounding factors. Optimum cutoff values were defined based on their highest diagnostic accuracy according to the *ROC* curves. *p* < 0.05 was considered statistically significant (∗*p* < 0.05, ∗∗*p* < *0.01*).

## Results

### Characteristics of the subjects

**Fig. 3:**
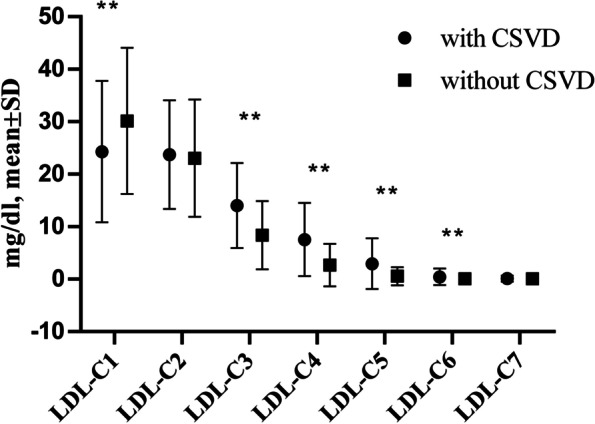
The level of LDL subtypes for patients with and without CSVD

Ultimately, 623 eligible patients were included in the present study. Of the 623 eligible patients, 487 were included in the CSVD group, and 136 were included in the group without CSVD (control group). The mean ages of the CSVD patients and controls were 65.13 ± 9.98 and 60.38 ± 9.31 years old, with male ratios of 53.39 and 42.65%, respectively. The baseline characteristics of both study groups are shown in Table 1. The age, pulse pressure difference, proportion of male patients, hypertension, diabetes, previous ischemic stroke, fasting plasma glucose (FPG), HbA1c, creatinine, UA, LDL-C, and Lp-PLA2 were significantly higher in the CSVD group than in the control group (*P* < 0.05). LDL-C1, LDL-C3, LDL-C4, LDL-C5 and LDL-C6 showed statistically significant differences between the groups, with *P* < 0.01 (Fig. 3). BMI, coronary artery disease, atrial fibrillation, previous cerebral hemorrhage, current smokers, current alcohol users, CRP, *D*-dimer levels, Lp(a), TG, TC and LDL-C7 did not differ between the two groups (*P* ≥ 0.05), while the HDL-C concentration was significantly higher in the control group (*P* < 0.05).

**Table 1:** Clinical Characteristics at Baseline (*n* = 623) for patients with and without CSVD

Variables	with CSVD(*n* = 487)	without CSVD *n* = 136)	*P* value
Age, y, mean ± SD	65.13 ± 9.98	60.38 ± 9.31	0.000
Male, n (%)	260(53.39)	58(42.65)	0.027
BMI, kg/m^2^, mean ± SD	26.82 ± 31.69	24.15 ± 3.08	0.309
Pulse pressure, mmHg, mean ± SD	51.67 ± 20.75	45.70 ± 21.47	0.004
Hypertension, n (%)	311(64.79)	53(39.55)	0.000
Diabetes, n (%)	109(22.71)	17(12.69)	0.011
Coronary artery disease, n (%)	38(7.92)	8(5.97)	0.449
Atrial fibrillation, n (%)	3(0.63)	0(0.00)	0.359
Previous cerebral hemorrhage, n (%)	15(3.13)	1(0.75)	0.126
Previous ischemic stroke, n (%)	179(37.29)	24(17.91)	0.000
Current smokers, n (%)	77(16.04)	20(14.93)	0.754
Current alcohol user, n (%)	65(13.54)	12(8.96)	0.156
CRP(mg/L), mean ± SD	4.06 ± 22.07	4.01 ± 17.03	0.981
*D*-dimer levels, mg/L, mean ± SD	0.55 ± 1.98	0.38 ± 0.42	0.369
FPG, mmol/L, mean ± SD	5.81 ± 2.09	5.34 ± 1.44	0.018
HbA1C, %, mean ± SD	5.99 ± 1.26	5.59 ± 0.88	0.002
creatinine, μmol /L, mean ± SD	64.95 ± 20.57	60.07 ± 14.28	0.011
Uric acid, μmol /L, mean ± SD	311.94 ± 85.93	294.61 ± 80.56	0.040
Lp(a), mg/L, mean ± SD	243.57 ± 230.55	272.18 ± 274.72	0.098
Total cholesterol, mean ± SD	4.40 ± 1.14	4.24 ± 1.17	0.136
Triglyceride, mean ± SD	1.98 ± 1.67	2.31 ± 1.96	0.062
LDL-C, mmol/L, mean ± SD	2.59 ± 0.84	2.39 ± 0.84	0.018
LDL-C1, mg/dl, mean ± SD	24.30 ± 13.48	30.14 ± 13.92	0.000
LDL-C2, mg/dl, mean ± SD	23.73 ± 10.35	23.04 ± 11.19	0.498
LDL-C3, mg/dl, mean ± SD	14.02 ± 8.11	8.40 ± 6.50	0.000
LDL-C4, mg/dl, mean ± SD	7.55 ± 6.99	2.69 ± 4.05	0.000
LDL-C5, mg/dl, mean ± SD	2.94 ± 4.82	0.56 ± 1.73	0.000
LDL-C6, mg/dl, mean ± SD	0.45 ± 1.57	0.08 ± 0.39	0.006
LDL-C7, mg/dl, mean ± SD	0.10 ± 0.57	0.06 ± 0.34	0.440
HDL-C, mmol/L, mean ± SD	1.12 ± 0.28	3.25 ± 16.12	0.005
Statin therapy, n (%)	242(50.42)	60(44.78)	0.248
Lipoprotein-associated phospholipase A2, ng/mL, mean ± SD	137.26 ± 23.83	140.26 ± 16.81	0.027

Continuous variables are shown as the mean ± standard deviation (SD), categorical variables are shown as numbers combined with percentage (%). Pulse pressure means the difference between the systolic and diastolic pressures

*BMI* Body mass index, *CRP* C-reactive protein, *FPG* fasting plasma glucose, *HbA1C* glycated hemoglobin, *LDL-C* indicates low-density lipoprotein cholesterol; Lp(a), lipoprotein(a)

### Relationship of LDL subtypes with CSVD risk

A logistic regression model was performed to analyze the relationship between LDL subtypes and CSVD risk. CSVD was used as a response variable, and risk factors were used as explanatory variables. Detailed results are shown in ​Table 2. LDL-C3 was significantly higher in the CSVD patients than in those without CSVD (OR (95% CI), 1.18(1.06–1.32), *P* < 0.05). There was no significant difference among the CSVD group and the controls in terms of LDL-C, LDL-C1, LDL-C2, LDL-C4, LDL-C5, LDL-C6, and LDL-C7 (*P* ≥ 0.05).

**Table 2:** The logistic regression analysis of LDL subtype for patients with and without CSVD

Variables	β	Wals	OR (95%CI)	*P* value
LDL-C1	0.010	0.646	1.01(0.99–1.03)	0.422
LDL-C2	− 0.047	3.801	0.95(0.91–1.00)	0.051
LDL-C3	0.169	8.680	1.18(1.06–1.32)	0.003
LDL-C4	−0.094	1.044	0.91(0.76–1.09)	0.307
LDL-C5	0.268	3.595	1.31(0.99–1.73)	0.058
LDL-C6	−0.102	0.120	0.90(0.51–1.60)	0.729
LDL-C7	−0.367	0.234	0.86(0.45–1.61)	0.628

Model 1 additionally adjusted for LDL subtypes

*OR* odds ratio, *CI* confidence interval

In addition, we made further adjustments for LDL subtypes, sex, age, pulse pressure, hypertension, previous ischemic stroke, diabetes and statin therapy in the multivariate logistic regression model (Table 3). LDL-C3 was still higher in the CSVD patients than in those without CSVD (OR (95% CI), 1.22(1.08–1.38), *P* < 0.05). Moreover, gender, age, hypertension and previous ischemic stroke showed statistically significant differences between the two groups (OR (95% CI), 0.62(0.40–0.99), 1.05(1.02–1.08), 2.04(1.26–3.31), and 1.60(0.85–3.00), respectively).

**Table 3:** The logistic regression analysis of LDL subtype for patients with and without CSVD

Variables	β	Wals	OR (95%CI)	*P* value
LDL-C1	0.033	5.934	1.03(1.01–1.06)	0.015
LDL-C2	−0.056	4.393	0.95(0.90–1.00)	0.036
LDL-C3	0.198	10.040	1.22(1.08–1.38)	0.002
LDL-C4	−0.088	0.792	0.92(0.75–1.11)	0.373
LDL-C5	0.291	3.736	1.34(1.00–1.80)	0.053
LDL-C6	−0.114	0.121	0.89(0.47–1.70)	0.728
LDL-C7	0.015	0.002	1.02(0.54–1.92)	0.964
Gender	−0.471	4.069	0.62(0.40–0.99)	0.044
Age	0.047	12.885	1.05(1.02–1.08)	0.000
Pulse pressure	0.008	1.904	1.01(1.00–1.02)	0.168
Hypertension	0.714	8.377	2.04(1.26–3.31)	0.004
Previous ischemic stroke	0.785	7.436	2.19(1.25–3.86)	0.006
Diabetes	0.469	2.138	1.60(0.85–3.00)	0.144
Statin therapy	−0.263	1.242	0.77(0.48–1.22)	0.265

Model 2 adjust for LDL subtypes, Gender, Age, Pulse pressure, Hypertension, Previous ischemic stroke, Diabetes and Statin therapy

*OR* odds ratio, *CI* confidence interval

### Correlation between clinical characteristics and CSVD risk

**Fig. 4:**
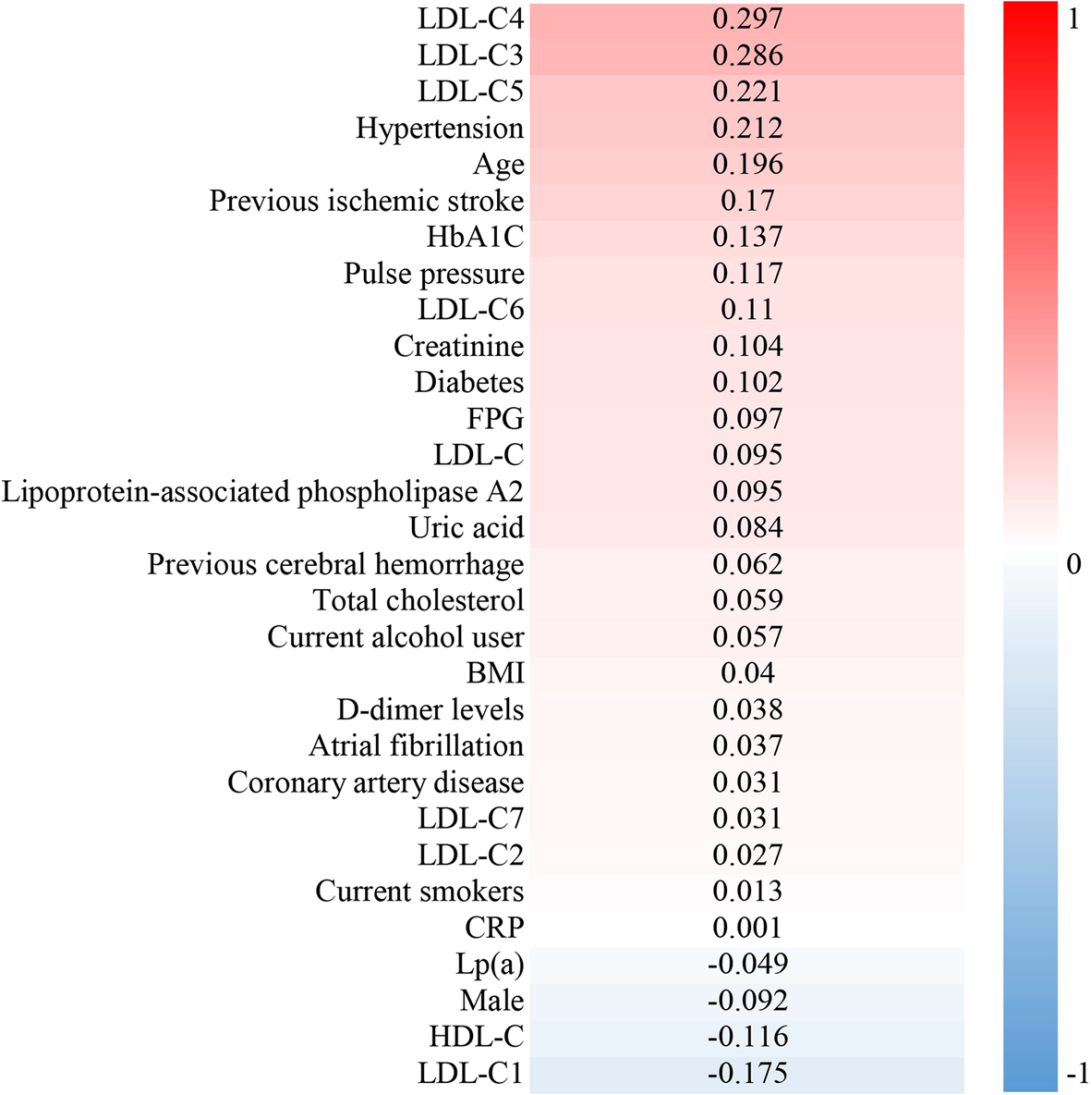
Heatmap showing the correlation between clinical characteristics and CSVD risk

The Pearson correlation coefficient was used to analyze the correlation between clinical characteristics and CSVD risk (Fig. 4). There was a positive correlation between the levels of LDL-C3, LDL-C4, LDL-C5, glucose, age, hypertension, previous ischemic stroke and CSVD risk (r > 0.15, *P* < 0.01). However, the LDL-C1 level, HDL and CSVD risk were negatively correlated (*r* = − 0.175, *r* = − 0.116, *P <* 0.01).

### Predictive value of LDL-C subtypes for CSVD risk

**Fig. 5:**
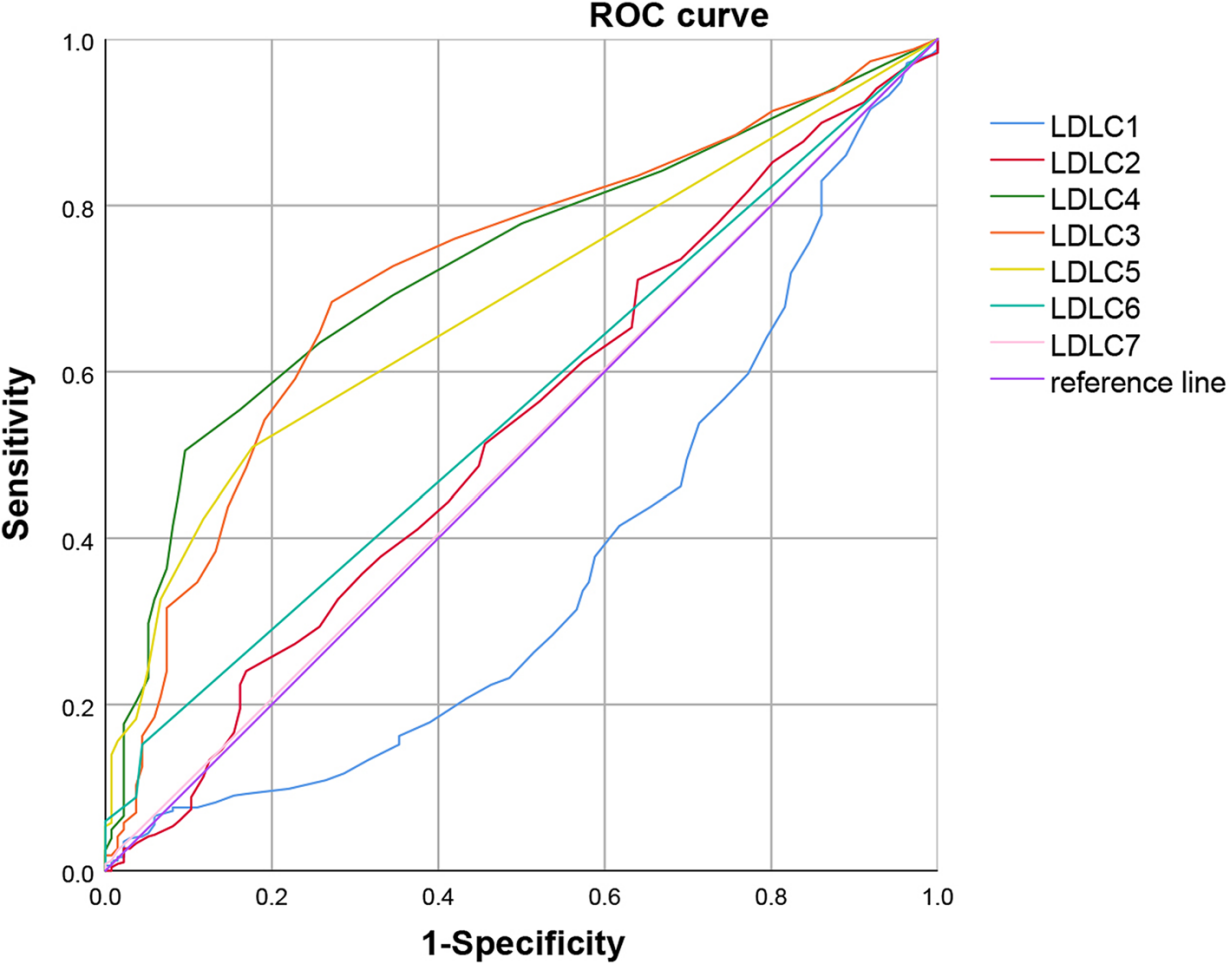
Predictive Value of LDL-C subtypes for CSVD Risk

To further evaluate the predictive values of LDL-C1, LDL-C2, LDL-C3, LDL-C4, LDL-C5, LDL-C6 and LDL-C7 in patients with CSVD, ROC curves and AUCs were created and depicted (Fig. 5, Table 4). LDL-C3 and LDL-C4 showed larger areas under the curves than the other subtypes of LDL-C (AUC, 0.718 and 0.730, respectively). Moreover, the best cutoff value of LDL-C3 to predict CSVD was 9.5 mg/dL with 68.4% sensitivity and 72.8% specificity, and the best cutoff value of LDL-C4 to predict CSVD was 5.5 mg/dL with 50.5% sensitivity and 90.4% specificity.

**Table 4:** The area under ROC curve

Variables	LDL-C	LDL-C1	LDL-C2	LDL-C3	LDL-C4	LDL-C5	LDL-C6	LDL-C7
Area	0.579	0.365	0.531	0.718	0.730	0.681	0.554	0.504

## Discussion

The main findings of this study were as follows: 1) even after adjustments for multiple confounding factors, including age, hypertension and previous ischemic stroke at baseline, higher baseline serum LDL-C3 levels were associated with an increased risk of CSVD; and 2) LDL-C3 and LDL-C4 can predict CSVD risk.

It is generally known that CSVD is a highly prevalent disease in older individuals. CSVD burden may signify a diminished capacity of cerebral tissue to withstand ischemia and could be a poor prognostic marker for stroke survivors. Therefore, there is continuous interest in identifying novel risk factors for CSVD burden. It is well known that age and hypertension are the most important risk factors for CSVD. Consistent with previous studies, we also found that age, hypertension and previous ischemic stroke were associated with the burden of CSVD in our study population.

Another important traditional risk factor is the elevation of LDL-C, and LDL-C is commonly present concurrently with relevant pathologies, including large artery atherosclerosis or CSVD. However, an increasing number of studies have found that a high proportion of patients with atherosclerosis or acute vascular events have plasma cholesterol and LDL-C levels within the normal range [34]. This shows that LDL-C levels may not fully reflect the biological effect of LDL and the real blood lipid level of patients.

Recently, studies have confirmed that LDL-C consists of a series of particles with different sizes, densities, physicochemical compositions, metabolic behaviors and atherogenicity [35]. Specifically, LDL-1 to LDL-7 were defined by gradient gel electrophoresis. Among them, LDL-3 to LDL-7 are also named small dense LDL (sdLDL-C) according to their small size and high density, whereas LDL-1 and LDL-2 are defined as large LDLs. According to the National Cholesterol Education Program Adult Treatment Panel III (NCEP III), a high level of sdLDL-C is an established risk factor for cardiovascular disease (CVD) [36], and sd-LDL is recognized as the most important vascular contributor [37]. However, their clinical significance for CSVD is unclear.

Although abnormalities in serum lipids and lipoproteins have long been identified as risk factors for CSVD, TC, TG, Lp(a) and LDL-C in both groups were within the normal reference range in both CSVD patients and controls, suggesting that they are not the best biomarkers for CSVD prediction. Multivariable logistic regression confirmed that LDL-C3 was an independent variable of CSVD after adjustment for traditional vascular risk factors. Additionally, the LDL-C3 and LDL-C4 AUCs for CSVD were superior to those of other lipids, which indicated that the levels of these two serum sdLDL-Cs had better risk prediction for CSVD than other lipids, including LDL-C. The mechanisms related to the atherogenicity of sdLDL-C have long been recognized and include penetrating the arterial wall more easily, being more susceptible to oxidation or binding to glycosaminoglycans in the arterial wall, having a decreased binding ability to LDL-C receptors, and possessing an increased plasma residence time; thus, several studies have stated that sdLDL-C is an independent and more potent risk factor for coronary and peripheral artery disease as well as carotid atherosclerosis than LDL-C [38, 39].

Additionally, the correlation of LDL subfractions and the relationship of sd-LDL and serum lipids were assessed in our study. Consistent with the lower distribution of LDL-1 in the CSVD group, LDL-1 was negatively correlated with the sdLDL-C particles LDL-3 to LDL-7, suggesting a lower potential atherogenicity of this particle. Considering the correlation coefficients of LDL-1 with LDL-C, it is highly recommended that not only LDL-C levels but also LDL subfractions (LDL-3 to LDL-7) should be taken into consideration for the management of CSVD. Generally, sdLDL-C is often accompanied by increased TG and TC and decreased HDL levels. In our study, there was a significant positive correlation between sdLDL-C levels and serum lipids, including TC, LDL-C, and TG, which is consistent with the findings reported by other studies [40].

Taken together, these observations may suggest that in patients with normal LDL-C levels, we need to consider other factors, such as sdLDL-C, to determine the risk of CSVD. Most importantly, a new prediction model was generated, which can help clinicians identify high-risk CSVD patients so that proper prevention measures can be taken to ease the potential burden and reduce suffering. Further, some evidence suggests that the presence and progression of cerebral atrophy is another potentially relevant manifestation of CSVD [41, 42], further study regarding the association between cerebral atrophy and sdLDL-C is warranted. Finally, whether neuropsychological alterations are a predictor of subcortical vascular dementia in the medium term and the relationship between sdLDL-C and silent lacunes on neuroimaging will be further explored in our future follow-up studies.

### Study strengths and limitations

This is the first report demonstrating that sdLDL-C is an independent risk factor for increased CSVD after additional adjustment for other traditional risk factors. However, there are some limitations in the current study. First, the sample size of the participants enrolled was relatively small. Second, because of the nature of the retrospective research design, incomplete data collection, and lack of follow-up of prognosis, only relevant conclusions can be drawn without mechanistic research. Third, this study involved individuals in the Han population in the same region. Fourth, the effects of inflammatory markers on atherosclerosis were not considered in this study. In addition, we only collected the history of statins, but not the history of fibrates and other lipid-lowering drugs. Finally, previous studies have revealed that half of the patients with a first-ever lacunar infarct have mild cognitive impairment with subcortical vascular features and its presence may be a predictor of subcortical vascular dementia in the medium-long term [43], we did not evaluate cognitive function in the present study. Therefore, a large sample and prospective research are needed to overcome the above limitations.

## Supplementary Information

**Additional file 1: Supplements Table 1.** The correlation between clinical characteristics and severity of CSVD. **Supplements Figure 1.** The level of LDL subtypes between different total CSVD scores. **Supplements Figure 2.** The distribution of each component (WMH, CMBs, EPVS, and lacunes) of CSVD.

## Data Availability

Study data are available from the corresponding author upon request.

## Abbreviations

AIS: Acute ischemic stroke
BMI: Body mass index
CMB: Lobar and deep cerebral microbleed
CNS: Central nervous system
Cr: Creatinine
CSF: Cerebrospinal fluid
CSVD: Cerebral small vascular disease
CVD: Cardiovascular disease
DWI: Diffusion weighted imaging
FBG: Fasting blood glucose
FLAIR: Fluid-attenuated inversion recovery
HbA1c: Glycosylated hemoglobin
H-CRP: High-sensitivity C-reactive protein
IMT: Intima–media thickness
LDL: Low-density lipoprotein
Lp(a): Lipoprotein a
Lp-PLA2: Lipoprotein-associated phospholipase A2
MRI: Magnetic resonance imaging
PVS: Perivascular space
sdLDL-C: Small and dense low-density lipoprotein cholesterol
SWI: Susceptibility-weighted imaging
TC: Total cholesterol
TG: Triglyceride
UA: Uric acid
WMH: White matter hyperintensity
AIS: Abbreviated Injury Score
NIHSS: NIH Stroke Scale
