# Supplementary material for: Association between small dense low-density lipoprotein cholesterol and neuroimaging markers of cerebral small vessel disease in middle-aged and elderly Chinese populations
Source: BMC Neurol. 2021 Nov 9;21:436. doi: 10.1186/s12883-021-02472-6 (PMC8576978; doi:10.1186/s12883-021-02472-6)

Supplements table 1. The correlation between clinical characteristics and severity of CSVD.

| Variables | LDL-C | LDL-C1 | LDL-C2 | LDL-C3 | LDL-C4 | LDL-C5 | LDL-C6 | LDL-C7 |
| --- | --- | --- | --- | --- | --- | --- | --- | --- |
| severity of CSVD, *r* | 0.151 | -0.207 | 0.056 | 0.367 | 0.405 | 0.385 | 0.201 | 0.061 |
| *P* | 0.000 | 0.000 | 0.163 | 0.000 | 0.000 | 0.000 | 0.000 | 0.126 |

*r*, Spearman correlation coefficient.

Supplements figure 1. The level of LDL subtypes between different total CSVD scores.


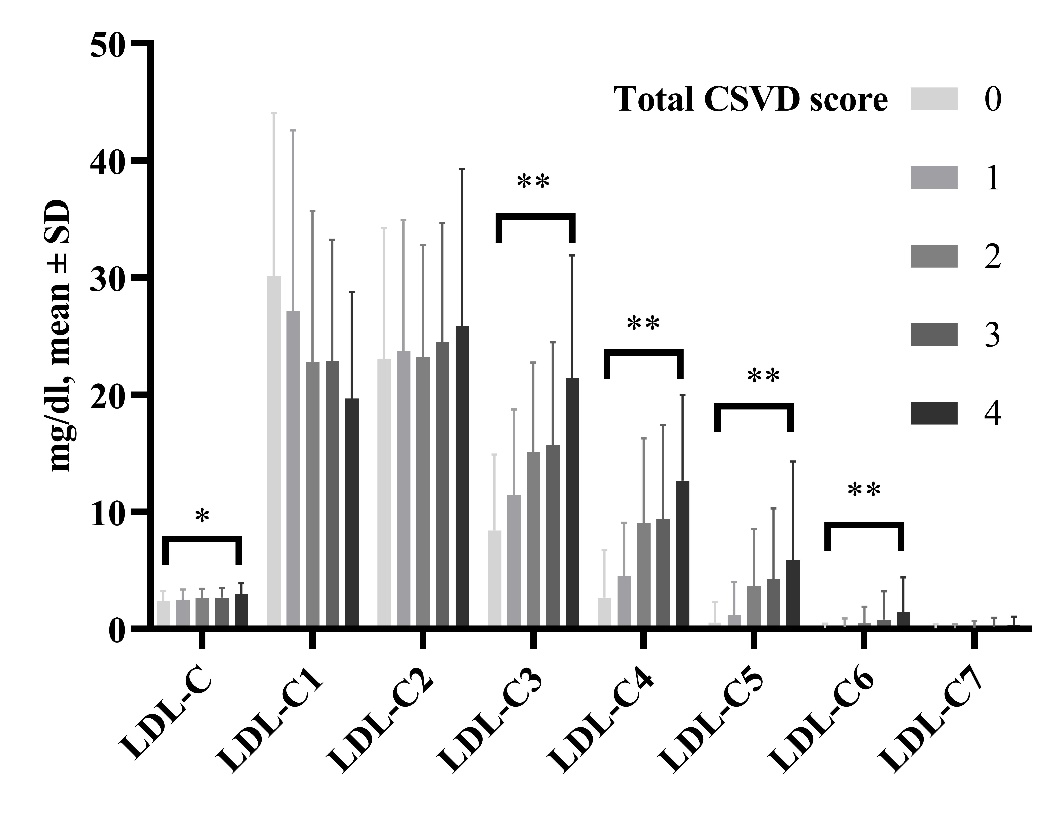


* *P*<0.05, ** *P*<0.01.

Supplements figure 2. The distribution of each component (WMH, CMBs, EPVS, and lacunes) of CSVD.


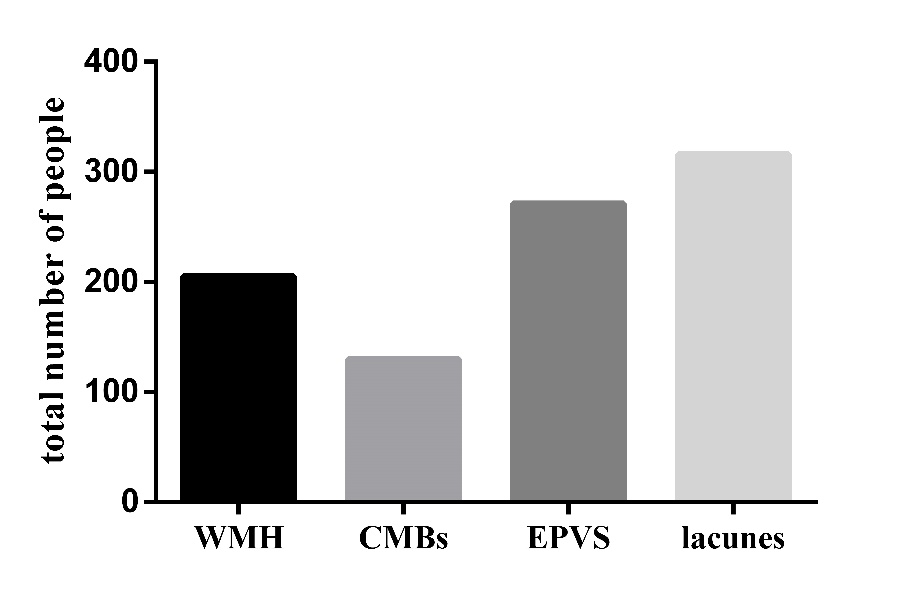

Supplement: Supplementary file 1 — Additional file 1: Supplements Table 1. The correlation between clinical characteristics and severity of CSVD. Supplements Figure 1. The level of LDL subtypes between different total CSVD scores. Supplements Figure 2. The distribution of each component (WMH, CMBs, EPVS, and lacunes) of CSVD. [file 12883_2021_2472_MOESM1_ESM.docx]
